# Pain Sensitivity Modifies Risk of Injury-Related Temporomandibular Disorder

**DOI:** 10.1177/0022034520913247

**Published:** 2020-03-20

**Authors:** S. Sharma, R. Ohrbach, R.B. Fillingim, J.D. Greenspan, G. Slade

**Affiliations:** 1 Department of Orofacial Pain and Jaw Function, Faculty of Odontology, Malmö University, Malmö, Skåne, Sweden; 2 Department of Oral Diagnostic Sciences, University at Buffalo School of Dental Medicine, Buffalo, NY, USA; 3 Department of Community Dentistry & Behavioral Science, College of Dentistry, University of Florida, Gainesville, FL, USA; 4 Department of Neural and Pain Sciences, and Brotman Facial Pain Clinic, University of Maryland School of Dentistry, Baltimore, MD, USA; 5 Division of Pediatric and Population Health, UNC Adams School of Dentistry, Chapel Hill, NC, USA; 6 Department of Epidemiology, University of North Carolina Gillings School of Global Public Health, Chapel Hill, NC, USA

**Keywords:** temporomandibular joint disorders, pain threshold, incidence, risk factors, prospective studies, central sensitization

## Abstract

This study evaluates contributions of jaw injury and experimental pain sensitivity to risk of developing painful temporomandibular disorder (TMD). Data were from the Orofacial Pain: Prospective Evaluation and Risk Assessment (OPPERA) nested case-control study of incident painful TMD. Injury and subsequent onset of painful TMD were monitored prospectively for ≤5 y in a community-based sample of 409 US adults who did not have TMD when enrolled. At baseline, thermal-pressure and pinprick pain sensitivity, as potential effect modifiers, were measured using quantitative sensory testing. During follow-up, jaw injury from any of 9 types of potentially traumatic events was determined using quarterly (3-monthly) health update questionnaires. Study examiners classified incident painful TMD, yielding 233 incident cases and 176 matched controls. Logistic regression models, estimated incidence odds ratios (IORs), and 95% confidence limits (CLs) were used for the association between injury and subsequent onset of painful TMD. During follow-up, 38.2% of incident cases and 13.1% of controls reported 1 or more injuries that were 4 times as likely to be intrinsic (i.e., sustained mouth opening or yawning) as extrinsic (e.g., dental visits, whiplash). Injuries due to extrinsic events (IOR = 7.6; 95% CL, 1.6–36.2), sustained opening (IOR = 5.4; 95% CL, 2.4–12.2), and yawning (IOR = 3.4; 95% CL, 1.6–7.3) were associated with increased TMD incidence. Both a single injury (IOR = 6.0; 95% CL, 2.9–12.4) and multiple injuries (IOR = 9.4; 95% CL, 3.4,25.6) predicted greater incidence of painful TMD than events perceived as noninjurious (IOR = 1.9; 95% CL, 1.1–3.4). Injury-associated risk of painful TMD was elevated in people with high sensitivity to heat pain (IOR = 7.4; 95% CL, 3.1–18.0) compared to people with low sensitivity to heat pain (IOR = 3.9; 95% CL, 1.7–8.4). Jaw injury was strongly associated with elevated painful TMD risk, and the risk was amplified in subjects who had enhanced sensitivity to heat pain at enrollment. Commonly occurring but seemingly innocuous events, such as yawning injury, should not be overlooked when judging prognostic importance of jaw injury.

## Introduction

Injury has a complex causal relationship to pain. Landmark prospective studies of back pain show that its incidence is influenced by multiple risk factors—for example, psychosocial status, job satisfaction, and compensation—in addition to the more proximal role of occupational injury (Bigos et al. 1992; Linton et al. 1994; Côté et al. 2008). Likewise, multiple factors in addition to motor vehicle collision (MVC) contribute to MVC-based neck pain disorders (McLean et al. 2011; McLean et al. 2014). While much less is known about the etiologic importance of musculoskeletal “strains” and “sprains” where no tissue damage is typically observable, experience from sports medicine suggests that all types of injury, with or without observable tissue damage, are equally challenging for clinical management of pain (Orchard and Best 2002; Bahr 2009). Better understanding of any injury-pain relationship requires careful methods.

One shortcoming in the evidence regarding causal effects of injury on pain is that most studies are cross-sectional: recall of injury is retrospective, and associations are prone to recall bias (Brown et al. 1998; Cote et al. 2000). Prospective cohort studies avoid such problems by establishing a temporal sequence between injury and development of subsequent pain disorder. A consistent finding from the few available prospective cohort studies is that relatively few individuals who experience injury develop a pain disorder (Hu et al. 2016; Sharma et al. 2019). A plausible explanation is that some individuals have preexisting traits that hamper recovery from injury, increasing the risk that the initial pain from injury will persist. Based on extensive preclinical and human studies, a very likely neural mechanism that can amplify effects of injury is central sensitization, defined as “amplification of central nervous system (CNS) neural signaling that elicits pain hypersensitivity” (Woolf 2011). However, we know of no prospective cohort studies that have attempted to assess the effects of a latent sensitized state for its potential to amplify the effect of injury on risk of developing clinical pain.

Temporomandibular disorders (TMDs) produce significant pain and limitation of jaw function, although, characteristically, there is no apparent trauma or pathology to account for the symptoms. Nonetheless, when queried, patients often cite a preexisting injury as a trigger of the symptoms, and retrospective studies report strong associations between history of injury and odds of TMD (Haggman-Henrikson et al. 2004; Klobas et al. 2004; Visscher et al. 2005; Caroll 2007; Grushka et al. 2007; Salè et al. 2010; Ohrbach et al. 2011). In addition to problems of recall bias, those retrospective studies usually inquire only about injuries from obvious trauma (e.g., following a blow to the face), overlooking potential injury from routine jaw function (e.g., prolonged mouth opening). Many studies of TMDs have other methodological limitations, including lack of suitable comparison groups (Martin et al. 2007; DeAngelis et al. 2009; Sahebi et al. 2010), use of comparison groups where TMD misclassification is likely (Klobas et al. 2004), and nonvalidated methods to diagnose TMD and evaluate injury (Huang et al. 2002; Martin et al. 2007; Sahebi et al. 2010; Salè et al. 2010).

This study investigated the incidence of both jaw injury and painful TMD prospectively to determine if risk of developing first-onset painful TMD is influenced by antecedent events of trauma or injury. We then evaluated antecedent measures of pain amplification as potential effect measure modifiers.

## Methods

### Study Population

Data were from the Orofacial Pain: Prospective Evaluation and Risk Assessment (OPPERA) nested case-control study of incident painful TMD (Slade, Bair, et al. 2011). As described in detail elsewhere (Slade, Bair, et al. 2011), the multisite study (Buffalo, New York; Chapel Hill, North Carolina; Baltimore, Maryland; Gainesville, Florida) enrolled individuals aged 18 to 44 y who had no significant history of facial pain, as based on no prior episodes of face pain sufficient to warrant a TMD diagnosis, and had no jaw injury in the 6 mo prior to enrollment (Bair et al. 2013). A history of jaw injury prior to 6 mo before enrollment did not exclude enrollment if there was no diagnosable painful TMD. Other eligibility criteria were based on specific health factors (Bair et al. 2013). Participants were followed for up to 5 y from 2006 to 2011, during which time examiners confirmed 260 incident cases of first-onset painful TMD. Index date for cases was when the participant received a clinical examination confirming the presence of first-onset painful TMD. Incident cases had to meet criteria for Research Diagnosic Criteria for TMD/TMD examination findings of myalgia or arthralgia (Bair et al. 2013). At the time of TMD onset, potential TMD-free controls were identified from among the cohort members based on matching variables of time since enrollment (from enrollment to index date), sex, and study site; the individual control was then selected at random. Index date was assigned to matched controls upon clinical examination, confirming the absence of TMD. Sixty-four of the potential controls did not return, leaving 196 selected controls without painful TMD when examined. Of the 260 cases and 196 controls, 47 participants (27 cases, 20 controls) were excluded from this analysis due to insufficient data about injury.

### Trauma Events and Jaw Injury Exposure

After enrollment, experience of potentially injurious events and perceived jaw injury was assessed at 3-monthly intervals using a quarterly health update (QHU) questionnaire. To enumerate events that had an “extrinsic” cause, participants were asked if they had experienced each of 7 potentially traumatic events: tooth extraction or dental treatments; motor vehicle accidents; accidents resulting in whiplash; oral intubation; sports injury including falls, bumps, and blows; injuries to the head; and injuries to the neck and shoulder region. For positive responses, an additional question asked if the event(s) had injured the jaw. Two additional questions asked about “intrinsic” injuries from each of 2 normal jaw functions: yawning and sustained mouth opening.

Three derived measures of exposure to traumatic events and/or injuries were then computed: 1) any jaw injury, a binary variable, signified 1 or more intrinsic or extrinsic injuries that occurred at any point during follow-up; 2) a composite indicator of events and injuries was classified using 4 mutually exclusive categories (no events, extrinsic events reported without injury, single injury reported in any 1 quarter, or multiple injuries reported in 2 or more quarters); and 3) types of injury were classified into 3 potentially overlapping categories (injuries due to extrinsic events, injuries due to yawning, and injuries due to prolonged opening) that were compared with a reference group reporting no injuries.

### Covariates

Potential confounders were selected based on published literature. Sociodemographic variables included age, sex (male or female), race/ethnicity (White, Black, Asian, Hispanic, and Other), marital status (married or living as married, divorced, separated or widowed, and never married), health insurance (yes or no), US lifetime residency (yes or no), education (high school or less, some college, college graduate, and postgraduate), annual household income (<$40,000, ≥$40,000, or not reported), and an 11-point rating of satisfaction with material financial status. Smoking history was classified as never smoker, former smoker, and current smoker. Height and weight recorded at enrollment were used to compute body mass index, analyzed as a continuous measure.

Psychological covariates were scale scores of depression and anxiety (Symptom Checklist–90 Revised [SCL-90R]; Derogatis 1994), posttraumatic stress disorder (PTSD) events and symptoms (Weathers et al. 1993), perceived stress (Perceived Stress Scale–10; Cohen et al. 1983), mood states (Profile of Mood States–Bipolar [PMOS-Bi]; Lorr et al. 1998), physical symptoms (Comprehensive Pain and Symptom Questionnaire [CPSQ]; Ohrbach et al. 2011), and coping (Coping Strategies Questionnaire–Revised; Riley and Robinson 1997); these variables are explained elsewhere (Fillingim et al. 2013).

Covariates reflecting clinical characteristics of the masticatory system were based on self-reported history of jaw injury prior to enrollment, prior facial pain, characteristic pain intensity, temporomandibular joint (TMJ) noise and TMJ locking, nonspecific jaw symptoms, and pain on jaw opening. Pain from clinical provocation by either mobility or palpation was also included. The Oral Behaviors Checklist (OBC) questionnaire (Markiewicz et al. 2006) assessed overuse of jaw. Note that reported history of TMJ noise or TMJ locking in the month prior to enrollment in the absence of clinically verified TMD was not a study exclusion criterion; such clinical phenomena were considered potential risk factors for developing a pain disorder.

Five measures of experimental pain sensitivity were considered potential effect measure modifiers. Each measure was a factor score derived using principal components analysis of 33 variables from quantitative sensory testing (QST) conducted at baseline; the raw variables were transformed to standard scores, with low values representing low pain sensitivity and high values representing high pain sensitivity, as described elsewhere (Greenspan et al. 2011). The factor scores were labeled 1) heat-pain ratings of suprathreshold stimuli and tolerance; 2) aftersensation ratings from heat; 3) mechanical cutaneous-pain ratings, temporal summation aftersensations, and threshold; 4) pressure pain thresholds; and 5) heat-pain temporal summation (Greenspan et al. 2011).

Reporting of this observational study conforms with the Strengthening the Reporting of Observational Studies in Epidemiology (STROBE) guidelines (von Elm et al. 2008).

### Statistical Analysis

When more than 10% of the values for a covariate were missing, a separate category was created while other missing values were excluded from the analysis. Descriptive statistics for baseline characteristics of cases and controls were generated. Binary logistic regression models were used to compute incidence odds ratios (IORs) and associated 95% confidence limits (CLs) as estimates of the association between jaw injury and painful TMD incidence. Incidence odds is the ratio of the number of people who developed TMD relative to the number who did not, while the IOR is the ratio of incidence odds in injured relative to noninjured groups. Because the study used frequency matching of cases and controls, unconditional logistic regression was used. Injury was used as the sole predictor to estimate the unadjusted association, whereas successive multivariable models estimated associations that were adjusted for 1) study site (accounting for site-specific variation), 2) study site and demographics (consistent with previous OPPERA analyses), and 3) study site, demographics, and other covariates that were found to be potential confounders. All analytic models were also adjusted for time since enrollment. The criteria for a potential confounding variable were nominal association (*P* < 0.2) with injury and TMD and when addition of the variable to the univariate model changed the incidence odds ratio by more than 10%. Misclassification bias with regard to missing injury reports was assessed by coding missing injury values as positive for injury. Potential additive effect modification due to pain sensitivity was evaluated by computing the relative excess risk for interaction (RERI), attributable proportion for interaction (AP), and synergy index (SI) and their associated 95% CLs and *P* values using methods described by Knol (Knol et al. 2007) and Hosmer (Hosmer and Lemeshow 1992). For stratified analysis of effect modification, the pain sensitivity measures were dichotomized using a median split.

### Sensitivity Analysis

Sensitivity analysis investigated attribution bias that potentially is created if events are spuriously reported as injurious because they coincide with jaw pain symptoms. Odds ratios were first estimated using concurrent reports of injury and pain within a single QHU. A second model used a lagged measure of injury, enumerating only injuries reported before the 3-mo period in which painful TMD symptoms developed. We reasoned that any difference in odds ratios would signify the degree of attribution bias, with the lagged measure representing the least biased measure. Due to clustering of repeated QHU data within individuals, logistic regression models were estimated using generalized estimated equations models.

## Results

### Descriptive

After excluding individuals without valid QHU or injury data, there were 233 incident TMD cases and 176 matched controls. Among cases, 8.1% experienced injury, with 1.2% to 2.8% reporting injury from extrinsic events. Greater percentages of cases experienced injury from yawning (22.3%) or sustained mouth opening (24.5%). One or more types of injury were reported by 38.2% of cases and 13.1% of controls (Table 1).

**Table 1.** Frequencies of Trauma Events and Injuries in Cases and Controls: OPPERA Nested Case-Control Study (*n* = 409).

	TMD Case Classification, No. (%)		
Characteristics	Incident Cases (*n* = 233)	Controls (*n* = 176)	*P* Value^a^
Type of extrinsic trauma event(s) associated with injury^b^			
None (reference)	214 (91.9)	174 (98.9)	
Any extrinsic	17 (7.3)	2 (1.4)	<0.01^c^
Whiplash	3 (1.2)	0	0.26^c^
Motor vehicle accident	5 (2.0)	0	0.07^c^
Tooth extraction/dental treatment	5 (2.0)	1 (0.5)	0.23^c^
Oral intubation	0	0	
Fall/bump/sports injury	7 (2.8)	1 (0.5)	0.08^c^
Injury to shoulder/neck	5 (2.0)	0	0.07
Injury affecting the head	5 (2.0)	1 (0.5)	0.23^c^
Type of intrinsic injury			
None (reference)	152 (65.2)	155 (88.1)	
Yawning injury	52 (22.3)	15 (8.5)	<0.01
Sustained mouth opening injury	57 (24.5)	12 (6.8)	<0.001
Any jaw injury			
No (reference)	144 (61.8)	153 (86.9)	
Yes	89 (38.2)	23 (13.1)	<0.001

OPPERA, Orofacial Pain: Prospective Evaluation and Risk Assessment; TMD, temporomandibular disorder.

a χ^2^ test for parametric categorical variables.

b Each type of injury is not mutually exclusive.

c Fisher’s exact test for nonparametric categorical variables.

Compared to controls, TMD cases were more likely to be lifetime US residents, be smokers, be less satisfied with material standards in life, have a different race profile (all *P* < 0.01), or be obese (*P* = 0.03). Cases also reported more nonspecific orofacial symptoms, jaw overuse behaviors, TMJ locking, and depression, anxiety, physical symptoms and sensations, stress, negative moods states, and greater sensitivity to heat-pain after sensations and tolerance (all *P* < 0.01) (Appendix Table).

### Any Jaw Injury

In the unadjusted model, the experience of any jaw injury was associated with a 4-fold increase in odds of incident TMD (IOR = 4.1; 95% CL, 2.5–6.9), and the relationship did not change appreciably after adjusting for time since enrollment, study site and demographics (IOR = 5.9; 95% CL, 3.3–10.6), and depression, the 1 covariate that met all criteria for confounding (IOR = 5.2; 95% CL, 2.9–9.5) (Table 2). When all potential confounders were used for adjustment, the point estimate was similar (IOR = 6.3; 95% CL, 3.2–12.3). When injury missing values were coded as injured, a moderate effect was maintained (IOR = 3.5; 95% CL, 2.1–6.0), whereas coding missing as not injured yielded an effect magnitude (IOR = 4.8; 95% CL, 2.7–8.6) that was very similar to the complete case analysis.

**Table 2.** Association between Number of Events and Type of Jaw Injury with Incident TMD: OPPERA Nested Case-Control Study (*n* = 409).

	TMD Case Classification, No. (%)		Incidence Odds Ratios (95% Confidence Limits)^a^
	Incident Cases	Controls	
Any injury	(*n* = 232)	(*n* = 175)	
No injury (reference)	144 (61.8)	153 (86.9)	1.0
Any injury	89 (38.2)	23 (13.1)	5.2 (2.9–9.5)
Composite events/injuries	(*n* = 232)	(*n* = 175)	
No events (reference)^b^	91 (39.2)	106 (60.6)	1.0
Trauma events without injury	52 (22.4)	46 (26.3)	1.9 (1.1–3.4)
Single injury^c^	58 (25.0)	16 (9.1)	6.0 (2.9–12.4)
Multiple injuries^d^	31 (13.4)	7 (4.0)	9.4 (3.4–25.6)
Type of jaw injury^e^	(*n* = 233)	(*n* = 176)	
No injury (reference)	144 (61.8)	153 (86.9)	1.0
Due to extrinsic events (e.g., MVC)	12 (5.2)	2 (1.14)	7.6 (1.6–36.2)
Due to yawning	36 (15.5)	14 (8.0)	3.4 (1.6–7.3)
Due to sustained mouth opening	40 (17.2)	11 (6.3)	5.4 (2.4–12.2)

MVC, motor vehicle collision; OPPERA, Orofacial Pain: Prospective Evaluation and Risk Assessment; TMD, temporomandibular disorder.

a Adjusted for time since enrollment, study site, demographics (age, race, and sex), and depression.

b Included individuals with no extrinsic event and no intrinsic types of injuries.

c Only 1 positive report of injury across the health update questionnaires (QHUs).

d More than 1 positive report for injury across the QHUs.

e Reporting specific type within and across QHUs.

### Composite Indicator of Events and Injuries

The extent of injury, ranging from events without injury to multiple injuries, was analyzed and compared to no trauma events. Events without injury (IOR = 1.9; 95% CL, 1.1–3.4) doubled the odds for developing painful TMD. However, the effect was much more pronounced for a single injury (IOR = 6.0; 95% CL, 2.9–12.4) and for injury on more than 1 occasion (IOR = 9.4; 95% CL, 3.4–25.6) (Table 2).

### Types of Jaw Injuries

The 3 types of trauma are presented in Table 2 as mutually exclusive; for example, the association between injury from yawning and incident painful TMD is independent from any other type of injury. A strong but imprecise association was seen between extrinsic injury and painful TMD (IOR = 7.6; 95% CL, 1.6–36.2). Injury due to sustained mouth opening showed almost twice as strong an association with painful TMD cases (IOR = 5.4; 95% CL, 2.4–12.2) as injuries due to yawning (IOR = 3.4; 95% CL, 1.6–7.3) (Table 2).

### Pain Sensitivity and Injury

Of the 5 measures of pain sensitivity, the factor scores for heat-pain aftersensations and tolerance showed evidence of effect measure modification (*P* < 0.10, Table 3), and the incidence odds ratio for association of any injury and TMD was greater for subjects with high sensitivity to heat-pain (IOR = 7.4; 95% CL, 3.1–18.0) than among individuals with low sensitivity to heat-pain (IOR = 3.9; 95% CL, 1.7–8.4) (Table 4). The RERI was 6.1 (95% CL, –2.7 to 14.9; *P* = 0.17), signifying that the combined effect of injury and heat-pain sensitivity exceeded the *sum* of the individual exposures. The AP among individuals jointly exposed was 59% (95% CL, 0.2–1.0; *P* = 0.004) and the SI was 2.9 (95% CL, 0.9–9.4; *P* = 0.08), signifying that the ratio between combined exposure and the sum of the individual exposures was greater than 1 (Table 4).

**Table 3.** Interaction between Injury and Quantitative Sensory Testing Component Score Measures (Continuous Variables).

	*P* Value for Interaction
Component 1 (heat-pain ratings)	0.45
Component 2 (heat-pain aftersensations)	0.05
Component 3 (mechanical cutaneous pain sensitivity)	0.21
Component 4 (pressure pain thresholds, reverse coded)	0.83
Component 5 (heat-pain temporal summation)	0.78

Logistic regression models adjusted for time since enrollment, study site, age, race, sex, and depression.

**Table 4.** Association between Jaw Injury, Heat Pain, and TMD: OPPERA Nested Case-Control Study (*n* = 409).

				Incidence Odds Ratio (95% CIs)^a^	
Heat-Pain Sensitivity^b^	Injury	Total No. (*n* = 409)	Incident Cases, No. (%) (*n* = 233)	Stratified Effect of Injury on TMD	Joint Effects of Injury and Pain Sensitivity on TMD
**Low (≤–0.15)**	No	155	71 (30.5)	1.0 (reference)	1.0 (reference)
	Yes	49	35 (15.0)	3.86 (1.78–8.40)	3.86 (1.77–8.40)
**High (>–0.15)**	No	142	73 (31.3)	1.0 (reference)	1.39 (0.85–2.28)
	Yes	63	54 (23.2)	7.44 (3.08–18.00)	10.37 (4.28–25.12)

Relative excess risk due to interaction (RERI) = 6.11 (95% CL, –2.67 to 14.90), *P* = 0.17. Attributable proportion to interaction (AP) = 0.59 (95% CL, 0.18–0.99), *P* = 0.004. Synergy index (SI) = 2.88 (95% CL, 0.88–9.37), *P* = 0.08.

OPPERA, Orofacial Pain: Prospective Evaluation and Risk Assessment; TMD, temporomandibular disorder.

a Adjusted for time since enrollment, study site, demographics (age, sex, and race), and depression.

b Pain sensitivity measure: quantitative sensory testing and heat-pain after sensations and tolerance.

### Sensitivity Analysis of Attribution Bias

In the sensitivity analysis of attribution bias, the concurrent model yielded a stronger association with painful TMD symptoms (IOR = 5.2; 95% CL, 3.6–7.6) than the lagged measure of exposure (IOR = 2.6; 95% CL, 1.6–4.1), although the latter remained statistically significant (Table 5).

**Table 5.** Association between Injury and Heat-Pain Sensitivity: Concurrent and Lagged Models.

	QHU (*n* = 1,932) Pain Threshold, No. (%)		
Model	Pain (*n* = 278)	No Pain (*n* = 1,654)	Incidence Odd Ratios (95% Confidence Limits)^a^
Concurrent			
No injury (reference)	197 (70.9)	1,544 (93.3)	1.0
Any injury	76 (27.3)	97 (5.9)	5.2 (3.6–7.6)
Lagged			
No injury (reference)	180 (64.8)	1,229 (74.3)	1.0
Any injury	38 (13.7)	80 (4.8)	2.6 (1.6–4.1)

QHU, quarterly health update (completed by 409 individuals).

a Computed using generalized estimating equations.

## Discussion

In this prospective cohort study of injury and painful TMD incidence, we found that self-reported jaw injury predicted a 5-fold increase in incidence odds of painful TMD after adjustment for potential confounders. This adds new evidence to our previous findings (Sharma et al. 2019) by singling out the types of injuries and traumatic events that contribute most to painful TMD incidence. Specifically, intrinsic injuries were far more common (yawning: 15.5% incident cases, 8.0% controls; sustained mouth opening: 17.2% incident cases, 6.3% controls) than extrinsic forms of injury (5.2% incident cases, 1.1% controls), and while extrinsic injuries had a more pronounced relative effect on incidence of painful TMD (IOR = 7.6; 95% CL, 1.6–36.2), intrinsic injuries from sustained mouth opening (IOR = 5.4; 95% CL, 2.4–12.2) and yawning (IOR = 3.4; 95% CL, 1.6–7.3) had surprisingly strong associations with painful TMD incidence. While sustained mouth opening is ubiquitous during dental and interventional medical treatments, among those who reported injury from sustained mouth opening, only a few (2.9%) reported injury from dental treatment, and no one reported injury from oral intubation. Consequently, relative to anecdotal reports in the literature, dental and medical treatments are infrequent contributors to injury from sustained mouth opening.

Another novel finding was that heat-pain sensitivity amplified the effect of injury on TMD incidence. Notably, heat-pain sensitivity was measured on the forearm, outside the trigeminal system, unlike TMD, and the injuries assessed here were all within the trigeminal system, suggesting several etiologically plausible explanations. One possibility is that greater heat-pain sensitivity is indicative of a generalized, low-grade hyperinflammatory phenotype (Slade, Conrad, et al. 2011) that amplifies the nociceptive processing system or prolongs the pain from injury due to persistence of nociceptive excitability. Alternatively, greater heat-pain sensitivity might signify weaker descending nociceptive inhibitory systems (Ossipov et al. 2014), rendering an injury more painful. Psychological mechanisms also warrant consideration: anxiety or psychological stress could be responsible for increased sensitivity to experimental heat-pain, and it might contribute to persistence of pain symptoms following injury.

A useful analog of the exposure measured in the present study comes from a rodent study (Hawkins and Durham 2016) in which sustained mouth opening led to increased levels of cytokines in both the peripheral trigeminal ganglion and the trigeminal nucleus caudalis. Elevated levels persisted beyond the rats’ return to normal nocifensive behavior. This is applicable to the current study for 2 reasons. First, a brief period of sustained, but presumably innocuous, jaw opening has demonstrable neurobiological consequences. The second is that those consequences set the stage for persistence of pain, evoking protective behaviors yet increasing vulnerability to further pain in that area. When interpreting the current findings, we regard heat-pain sensitivity at baseline as an indicator of either peripheral or central sensitization (Arendt-Nielsen et al. 2018), which could increase awareness of injury. We further speculate that the Calcitonin gene-related peptide-cytokine effects potentially induced by jaw opening injury may be amplified by the high pain sensitivity phenotype, which represents a latent sensitized or primed state of the peripheral and central nociceptive neurons that can be more easily activated by subsequent injury.

Strengths of this nested case-control study include minimized selection bias and recall bias compared to retrospective studies and the finding that no more than half of the effect of injury on TMD incidence is due to potential attribution bias. Similarly, it is noteworthy that, even among people who developed painful TMD, only a third reported injury; clearly, injury is not necessary for the same type of symptoms to emerge and lead to a diagnosis of painful TMD. Misclassification bias due to problems with self-reported exposure status, either as a function of possible limitations with the self-report instrument or of respondent behavior, did not affect the observed associations. Results support standard clinical practice of relying on self-report of prior injury as part of the medical history, with caveat of timing of symptom development.

Limitations of this study included absence of assessing injury specifically from each extrinsic event, reports of corresponding events associated with sustained mouth opening were not collected, and severity of each injury was not assessed. Also, it is possible that repeated assessments may attune one to marginal injury and hence overestimate the rate of injury as a source of bias. However, we expect that any attunement to marginal injury was nondifferential between cases and controls, which may have biased the point estimate toward the null, that is, we may have underestimated the magnitude of association between injury and TMD in our study.

In summary, there was a pronounced influence of jaw injury on painful TMD incidence. Seemingly innocuous injuries from yawning and sustained mouth opening likewise contributed to painful TMD and should be considered when evaluating the etiology of TMD pain. Moreover, effects of injury on painful TMD were amplified in individuals with heightened baseline pain sensitivity.

## Author Contributions

S. Sharma, contributed to conception, design, data analysis, and interpretation, drafted and critically revised the manuscript; R. Ohrbach, G. Slade, contributed to conception, design, data acquisition, analysis, and interpretation, drafted and critically revised the manuscript; R.B. Fillingim, J.D. Greenspan, contributed to conception and data acquisition, critically revised the manuscript. All authors gave final approval and agree to be accountable for all aspects of the work.

## Supplemental Material

DS_10.1177_0022034520913247 – Supplemental material for Pain Sensitivity Modifies Risk of Injury-Related Temporomandibular Disorder Click here for additional data file. Supplemental material, DS_10.1177_0022034520913247 for Pain Sensitivity Modifies Risk of Injury-Related Temporomandibular Disorder by S. Sharma, R. Ohrbach, R.B. Fillingim, J.D. Greenspan and G. Slade in Journal of Dental Research
