# Supplementary material for: Pain Sensitivity Modifies Risk of Injury-Related Temporomandibular Disorder
Source: J Dent Res. 2020 Mar 20;99(5):530–6. doi: 10.1177/0022034520913247 (PMC7174801; doi:10.1177/0022034520913247)
Supplement: DS_10.1177_0022034520913247 – Supplemental material for Pain Sensitivity Modifies Risk of Injury-Related Temporomandibular Disorder [file DS_10.1177_0022034520913247.pdf]

Pain Sensitivity Modifies Risk of Injury-Related Temporomandibular Disorder

S. Sharma, R. Ohrbach, R.B. Fillingim, J.D. Greenspan, and G. Slade

Appendix

Appendix Table. Baseline characteristics of participants: OPPERA nested case control study (n=409).

	**TMD case-classification**		
**Characteristic**	**Incident case (n=233)**	**Control (n=176)**	**p-value***
**Study site** (n (%))			
Baltimore, MD	53 (22.8)	48 (27.3)	0.51
Buffalo, NY	63 (27.0)	42 (23.9)	
Chapel Hill, NC	24 (10.3)	23 (13.1)	
Gainesville, FL	93 (39.9)	63 (35.8)	
**Age, yrs** (mean (sd))	28.9 (8.4)	28.6 (8.3)	0.70
**Gender** (n (%))			
Males	79 (33.9)	63 (35.8)	0.69
Females	154 (66.1)	113 (64.2)	
**Race** (n (%))			
White	124 (53.2)	87 (49.4)	<0.01†
Black/African American	77 (33.1)	45 (25.6)	
Asian	9 (3.9)	23 (13.1)	
Hispanic	16 (6.9)	17 (9.7)	
Other	7 (3.0)	4 (2.3)	
**Life time residency in U.S** (n (%))			
No	15 (6.4)	37 (21.0)	<0.01
Yes	215 (92.3)	138 (78.4)	
**Education** (n (%))			
Some college or less	146 (62.7)	93 (52.8)	0.05
College graduate or more	82 (35.2)	78 (44.3)	
**Family annual household Income (USD 1000s)** (n (%))			
<40	92 (39.5)	57(32.4)	0.32
≥40	100 (42.9)	82 (46.6)	
Not Stated	41 (17.6)	37(21.0)	
**Covered by health insurance** (n (%))			
No	35 (15.0)	22 (12.5)	0.50
Yes	189 (81.1)	145 (82.4)	
**Rating of satisfaction from material standards in life** (n (%))			
Low (0-5)	153 (65.7)	92 (52.3)	0.01
Mid (6-8)	64 (27.5)	62 (35.2)	
High (9-10)	12 (5.2)	18 (10.2)	
**Rating of satisfaction financial situation** (n (%))			
Low (0-3)	30 (12.9)	9 (5.1)	0.02

	TMD case-classification		
**Characteristic**	**Incident case (n=233)**	**Control (n=176)**	**p-value***
Mid (4-6)	68 (29.2)	49 (27.8)	
High (7-10)	130 (55.8)	114 (64.8)	
**Marital Status** (n (%))			
Married/Living as married	57 (24.5)	42 (23.9)	0.44
Divorced/Separated / Widowed	26 (11.2)	13 (7.4)	
Never Married	149 (64.0)	118 (67.1)	
**Smoking** (n (%))			
Never	144 (61.8)	140 (79.6)	<0.01
Current	48 (20.6)	20 (11.4)	
Former	32 (13.7)	10 (5.7)	
**Prior injury/event** (n (%))			
No	189 (81.1)	153 (86.9)	0.79
Yes	23 (9.9)	17 (9.7)	
**Prior facial pain** (n (%))			
No	169 (72.5)	138 (78.4)	0.17
Yes	61 (26.2)	36 (20.5)	
**Pain on jaw opening** (n (%))			
None	88 (37.8)	70 (39.8)	0.56
Any	143 (61.4)	101 (57.4)	
**Pain from palpation (masticatory)** (n (%))			
None	79 (33.9)	78 (44.3)	0.04
Any	153 (65.7)	98 (55.7)	
**Pain from palpation (body)** (n (%))			
None	84 (36.1)	74 (42.1)	0.22
Any	149 (64.0)	102 (58.0)	
**Pain from palpation (neck)** (n (%))			
None	133 (57.1)	114 (64.8)	0.12
Any	100 (42.9)	62 (35.3)	
**Facial Graded Chronic Pain Status** (n (%))			
No pain	182 (78.1)	152 (86.4)	0.16†
Pain	39 (16.7)	21 (11.9)	
Pain related disability	5 (2.2)	1 (0.6)	
**Facial Characteristic Pain Intensity** (mean(±SD))			
None	184 (79.0)	152 (86.4)	0.06
Any	45 (19.3)	22 (12.5)	
**No. of nonspecific orofacial symptoms** (n (%))			
None	153 (65.7)	145 (82.4)	<0.01
Any	79 (33.9)	31 (17.6)	
**Oral Behavior Checklist sum score** (mean (±SD))	23.39 (10.43)	20.07 (8.19)	<0.01
**TMJ noises & clicking** (n (%))			
No	144 (61.8)	124 (70.5)	0.08
Yes	82 (35.2)	48 (27.3)	

	TMD case-classification		
**Characteristic**	**Incident case (n=233)**	**Control (n=176)**	**p-value***
**TMJ locking** (n (%))			
No	189 (81.1)	158 (89.8)	0.01
Yes	39 (16.7)	15 (8.5)	
**BMI** (mean (±SD))	27.2 (6.8)	25.9 (6.1)	0.05
**SCL 90R Depression** (mean (±SD))	0.5 (0.5)	0.3 (0.4)	<0.01
**SCL 90R Anxiety** (mean (±SD))	0.3 (0.5)	0.1 (0.3)	<0.01
**Physical sxs: PILL** (mean (±SD))	98.1 (26.1)	87.5 (19.8)	<0.01
**PCS coping** (mean (±SD))	10.6 (10.4)	9.3 (8.5)	0.18
**Perceived stress scale** (mean (±SD))	15.9 (6.5)	13.3 (5.6)	<0.01
**PTSD/Number of Events** (n (%))			
None	118 (50.6)	107 (60.8)	0.04
Any	115 (49.4)	69 (39.2)	
**PTSD Symptoms** (mean (±SD))	29.9 (11.6)	25.8 (6.7)	0.02
**Mood state (POMS)** (mean (±SD))			
**Bi: Positive Affect**	84.1 (16.2)	88.4 (15.3)	<0.01
**Bi: Negative Affect**	52.5 (16.7)	47.8 (15.5)	<0.01
**Quantitative Sensory Test** (mean (±SD))			
**Heat pain ratings**	-0.004 (1.01)	-0.03 (1.00)	0.81
**Heat pain aftersensation**	0.2 (1.0)	-0.06 (0.8)	<0.01
**Mechanical cutaneous pain sensitivity**	-0.06 (0.89)	0.02 (1.13)	0.43
**Pressure pain threshold** (reverse coded)	0.06 (0.93)	0.03 (1.02)	0.75
**Heat pain temporal summation**	0.09 (1.06)	0.10 (0.93)	0.97

*χ^2 test for categorical variables and t-test for continuous variables
†Fisher's exact test for categorical variables and Wilcoxon-test for continuous variables
